# A Diagnostic Model for Dementia in Clinical Practice—Case Methodology Assisting Dementia Diagnosis

**DOI:** 10.3390/diagnostics5020113

**Published:** 2015-04-02

**Authors:** Elisabet Londos

**Affiliations:** Clinical Memory Reseach Unit, Institution of Clinical Sciences Malmö, Lund University, 20502 Malmö, Sweden; E-Mail: elisabet.londos@skane.se; Tel.: +46-40-335517; Fax: +46-40-335642

**Keywords:** diagnosis, dementia, case methodology, model, hypothesis

## Abstract

Dementia diagnosis is important for many different reasons. Firstly, to separate dementia, or major neurocognitive disorder, from MCI (mild cognitive impairment), mild neurocognitive disorder. Secondly, to define the specific underlying brain disorder to aid treatment, prognosis and decisions regarding care needs and assistance. The diagnostic method of dementias is a puzzle of different data pieces to be fitted together in the best possible way to reach a clinical diagnosis. Using a modified case methodology concept, risk factors affecting cognitive reserve and symptoms constituting the basis of the brain damage hypothesis, can be visualized, balanced and reflected against test results as well as structural and biochemical markers. The model’s origin is the case method initially described in Harvard business school, here modified to serve dementia diagnostics.

## 1. Introduction

Dementia or major neurocognitive disorder is a growing issue with an increasing number of sufferers. An European Union review estimated that 6.3 million individuals were affected in Europe 2010 [1]. Alzheimer’s disease (AD) is the most common neurocognitive disorder, followed by Lewy body and vascular disorders [2]. In the oldest old (85+), the mixed forms are the most prevalent [3]. Among people younger than 65 years at the time of onset of the neurocognitive disorder, a greater proportion has frontotemporal neurocognitive disorders compared to older age groups [4]. Accurate diagnosis is important for the treatment of the patient. The current search for a curative treatment of neurodegenerative disorders parallels the search for ways to diagnose the disorders as early as possible. In AD the CSF (cerebrospinal fluid) markers tau and beta-amyloid and PET (positron emission tomography) with ligands for amyloid and tau mirrors plaques and tangles and are the closest to the actual pathology that we get and this helps specific dementia diagnostics. Treatment today is however symptomatic and not related to the specific neuropathological findings, but rather related to disturbance of certain neurotransmitter producing nuclei such as nucleus basalis of Meynert (acetylcholine), locus coeruleus (noradrenaline), substantia nigra (dopamine) and raphe nuclei (serotonin), or NMDA (*N*-metyl-d-Aspartat) receptor antagonism to reduce extraneuronal glutamate levels. Nevertheless, individuals with more or less the same type and intensity of neurobiological changes can have different clinical presentations [5,6,7,8]. The cognitive reserve theory is increasingly used to at least partly explain this [9]. This theory implies that factors such as e.g., education, occupational level and life style habits may counteract the cognitive aging process and postpone “dementia onset”.

As practicing clinicians we make diagnoses taking many different pieces of information into account, such as cognitive, behavioral and psychiatric symptoms, description of disease onset and development, cognitive test results, imaging data, clinical criteria, laboratory findings and ability to manage activities of daily living.

To be comparable for research, the diagnoses have to rely on internationally accepted clinical criteria. The DSM (Diagnostic and Statistical Manual of Mental Disorders) and the ICD (International Classification of Diseases) criteria sets are the most commonly used, although different additional criteria, clinical or for research use, exists. The revised NINCDS-ADRDA (National Institute of Neurological and Communicative Disorders and Stroke and the Alzheimer’s Disease and Related Disorders Association) research criteria for Alzheimer’s disease incorporate biomarker findings which increases specificity for the diagnosis [10]. The DSMV uses a terminology of “neurocognitive disorder” instead of dementia and separates mild (former mild cognitive impairment, MCI) and major (former dementia) forms of the different underlying disorders (Alzheimer, Vascular, Lewy body, Frontotemporal, *etc.*) [11]. The concept major neurocognitive disorder used in DSMV is broader than dementia and encompasses disorders in which the primary clinical deficit is in cognitive function. The criteria for the various neurocognitive disorders are all based on defined cognitive domains.

No individual test or investigation can give the specific neurocognitive diagnosis in isolation. Instead, facts and findings have to be evaluated and weighted in the diagnostic process.

The following is a suggestion of a method developed in clinical practice, initially in the teaching of medical students, and later implemented in regular clinical practice, to facilitate the neurocognitive diagnostic process.

It is inspired by a variant of the case method originally developed at Harvard business school [12]. In the original method the concept is to put the student into the role of people facing the difficult decisions, inside perspective, while the actual variant of the case methodology tries to keep all possible solutions open to discussion from an outside perspective.

In the diagnostic case method the different aspects of the case are written on the board and variables can be considered and weighted against each other before the decision is made. An advantage is that with this approach it is easier to involve and analyze all possible factors, instead of getting too influenced by single investigational results such as imaging (CT/MRI), CSF biomarkers or cognitive test levels, especially since these are not the foundation of the existing clinical criteria.

## 2. Methods

In the practical application of the method three headlines are used: (a) Facts (b) Symptoms and (c) Investigational findings.

Under “Facts”, data from the patient such as age, gender, living situation (alone?), family, education, occupation, interests, heredity factors, other diseases (hypertension, diabetes, others), medications, pain, brain injury, infections, alcohol consumption *etc.* are listed.

The purpose of this section is to establish the cognitive reserve in the patient (demographic data, risk and preventive factors).

Under “Symptoms” the cognitive, behavioral, psychiatric and other clinically important symptoms, are listed. Examples; memory dysfunction and type of onset, visuospatial disturbance, dyspraxia, dysphasia, dysexecution, hallucinations, depression, motor symptoms, loss of insight, *etc.* These symptoms are thereafter used to form a crude brain damage hypothesis. For clarity, a simplified model of the brain is drawn and the location(s) of the symptoms are marked.

It is important to make the clinical hypothesis before the different test- and imaging results are introduced.

After that “Investigational findings”—results of cognitive tests, imaging, CSF—are listed and evaluated according to if they do or do not support the brain damage hypothesis.

The combined symptomatic/investigational hypothesis could then be compared with the descriptive clinical criteria of the different neurocognitive disorders. A decision about whether the disturbance is minor or major is also made with help from information of whether reduced independence in daily life is due to the cognitive symptoms.

## 3. Results and Discussion

### 3.1. Example 1 (Table 1)

**Table 1:** Example 1.

Facts	Symptoms	Investigational Findings
Woman, 81 years Widow, living alone, 2 children Heredity: mother and aunt developed dementia late in life 7 years of schooling Worked part-time in a shop Hypertension (HT) Diabetes (DM)	Insidious memory problems Stopped baking (iADL) The home is not tidy any more (iADL) Forgets to buy food (iADL) Weight loss Depressed Loss of initiative Incontinent	MMSE: 21/30p. Profile: orientation 6/10 delayed recall 1/3, attention (calculation) 3/5, pentagons 0/1 Cube: some 3-dimentionality but not correct Clock: puts the hands on 10 and 11 TMT A and B: slow CT: atrophy of the MTA bilaterally, periventricular, partly confluating white matter changes CSF: slightly elevated tau and p-tau, low beta amyloid

HT = hypertension; DM = diabetes mellitus; iADL = instrumental activities of daily living; MMSE = Mini mental state examination; TMT = Trail making test; CT = computerized tomography; MTA = medial temporal atrophy; CSF = cerebrospinal fluid.

#### Evaluation

Under “Facts” several risk factors for neurocognitive disorders are identified—DM, HT, heredity, loneliness, low schooling background—leading to a hypothesis of a compromised (low) cognitive reserve.

The “Symptoms” column give rise to the brain damage hypothesis with symptom localization in temporal, parietal and frontal areas.

Hypothesis: Alzheimer and subcortical vascular components.

Evaluating symptoms and hypothesis against “Investigational findings”: The profile of MMSE (Mini mental state examination) indicates temporal deficits (memory and orientation) which are confirmed by the CT MTA (medial temporal lobe atrophy). TMT (Trail making test) indicates mental slowness which could reflect the white matter changes on CT. Clock test, cube and pentagons indicate parietal engagement, which is supported by the reduced instrumental (iADL) ability in practical life.

The investigation supports a diagnosis of mixed Alzheimer and subcortical white matter disease. The components are evaluated as equally contributing to the cognitive disorder.

Clinical criteria for Alzheimer’s disease are fulfilled. The degree of neurocognitive impairment is regarded as major since the patient cannot manage her ordinary life independently.

### 3.2. Example 2 (Table 2)

**Table 2:** Example 2.

Facts	Symptoms	Investigational Findings
Man 69 years Female partner, 2 sons from an earlier relationship Retired sea captain 7 years schooling No specific interests except watching TV Coronary bypass operation at the age of 54 Hypertension Gastritis 15–20 standard glasses of alcohol per week	Memory problems last 2 years Motor and cognitive slowness Isolation Apathy with reduced interest for others Depression	MMSE: 16/30p. Profile: 4/10 orientation, 1/5 attention (calculation), 0/3 delayed recall, 2/3 in 3-stage command TMT A and B: slow Clock test: correct GDS: 10p CT: cerebellar atrophy, moderate frontal white matter hypodensities CSF: beta-amyloid mildly reduced, elevated neurofilaments, t-tau and p-tau slightly increased Lab: P-Eth and homocystein: elevated BP: 180/90

MMSE = Mini mental state examination; TMT = Trailmaking test; GDS = Geriatric depression scale; CT = computerised tomography; CSF = cerebrospinal fluid; P-Eth = Plasma ethanol; BP = blood pressure.

#### Evaluation

Under “Facts” vascular risk factors and complications from alcohol overconsumption (>14 standard glasses per week) compromise the cognitive reserve. Balancing this is younger age and social network which increase the brain reserve.

The “Symptoms” column leads to a brain damage hypothesis of temporal and frontosubcortical location, with emphasis on the frontosubcortical symptoms.

### 3.3. Discussion

Reduced cognitive function possibly due to alcohol overconsumption and depression. A vascular component is present on CT and reinforced by increased Neurofilament, but additional smaller Alzheimer changes cannot be excluded.

The underlying pathology is probably enhanced by the reduced brain reserve evaluated from lower schooling, hypertension and alcohol overconsumption, B12 and folate deficiency.

Reducing alcohol consumption may improve cognition and postpone additional neurocognitive signs.

The incongruence of 16 points on MMSE and a perfect clock indicated possibilities for improvement.

Treatment: reduced alcohol consumption, B12 and folate substitution, BP measurements. Follow-up with focus on AD signs.

The low result in MMSE was partly due to resignation and was improved to 26p after 6 months of alcohol cessation. The degree of neurocognitive impairment was then regarded as minor.

## 4. Conclusions

The modified case method described visualizes, in a structured way, the important different clinical factors needed to be taken into account in the diagnostic process of dementia. Clearly identifying the affected cognitive domains also supports the use of clinical criteria like DSMV. It is also highlights the importance to find as many additional factors as possible to be able to give an individually tailored treatment. The different treatment possibilities will probably increase in the future with improved understanding of the different inflammatory, vascular and neurodegenerative and other processes and their interaction. This model can help prepare putting together the diagnostic puzzle in a structured way, as well as weighing different possible treatable factors against each other in absence of a curable treatment based on the underlying neuropathology.

## References

[1] Wittchen H.U., Jacobi F., Rehm J., Gustavsson A., Svensson M., Jonsson B., Olesen J., Allgulander C., Alonso J., Faravelli C. (2011). The size and burden of mental disorders and other disorders of the brain in Europe 2010. Eur. Neuropsychopharmacol..

[2] Picard C., Pasquier F., Martinaud O., Hannequin D., Godefroy O. (2011). Early onset dementia: Characteristics in a large cohort from academic memory clinics. Alzheimer Dis. Assoc. Disord..

[3] Neuropathology Group. Medical Research Council Cognitive Function and Aging Study (2001). Pathological correlates of late-onset dementia in a multicentre, community-based population in England and Wales. Neuropathology Group of the Medical Research Council Cognitive Function and Ageing Study (MRC CFAS). Lancet.

[4] Perry D.C., Miller B.L. (2013). Frontotemporal dementia. Semin. Neurol..

[5] Wolk D.A. (2013). Amyloid imaging in atypical presentations of Alzheimer’s disease. Curr. Neurol. Neurosci. Rep..

[6] Duker A.P., Espay A.J., Wszolek Z.K., Rademakers R., Dickson D.W., Kelley B.J. (2012). Atypical motor and behavioral presentations of Alzheimer disease: A case-based approach. Neurologist.

[7] Grossman M. (2010). Primary progressive aphasia: clinicopathological correlations. Nat. Rev. Neurol..

[8] Bower J.H., Dickson D.W., Taylor L., Maraganore D.M., Rocca W.A. (2002). Clinical correlates of the pathology underlying parkinsonism: A population perspective. Mov. Disord..

[9] Whalley L.J., Deary I.J., Appleton C.L., Starr J.M. (2004). Cognitive reserve and the neurobiology of cognitive aging. Ageing Res. Rev..

[10] Dubois B., Feldman H.H., Jacova C., Dekosky S.T., Barberger-Gateau P., Cummings J., Delacourte A., Galasko D., Gauthier S., Jicha G. (2007). Research criteria for the diagnosis of Alzheimer’s disease: Revising the NINCDS-ADRDA criteria. Lancet Neurol..

[11] American Psychiatric Association (2013). Diagnostic and Statistical Manual of Mental Disorders: DSM 5.

[12] Greenhalgh A.M. (2007). Case Method Teaching as Science and Art: A Metaphoric Approach and Curricular Application. J. Manag. Educ..

